# Microbiology culture results in high risk foot clinic patients: an audit

**DOI:** 10.1186/1757-1146-4-S1-O50

**Published:** 2011-05-20

**Authors:** Jessica M White, Lynne Toh, Hugh G Dickson, Erika N Koo, Gentry S Winters, Marion G Harpur, John Widdup, Namson S Lau

**Affiliations:** 1 Department of Podiatry, Liverpool Hospital, Locked Bag 7103, Liverpool, NSW, 2170, Australia; 2 Department of Podiatry, La Trobe University, Melbourne, Victoria, 3086, Australia; 3 Department of Ambulatory Care, Liverpool Hospital, Locked Bag 7103, Liverpool, NSW, 2170, Australia

## Background

The majority of the clinical work of a high risk foot service is involved in the care of patients with diabetic foot ulcers. Infection is common, with the percentage of patients in a session receiving antibiotics ranging from 50-100%. We conducted an audit of microbiology culture results for our patients over a one-year period.

## Methods

Three reviewers extracted all microbiology results for all patients who had a current foot progress chart and who had been attending the high risk foot unit at Liverpool Hospital from the period of 15 September 2009 to 15 September 2010. The results were collated and entered into an Excel^©^ spread sheet. Fields included wound location, microbes, colonisation, and antibiotic susceptibility.

## Results

The total number of patients fitting the selection criteria was 131. The most common finding was coliform colonisation, in 83 patients (63.4%), followed by staphylococcus aureus, in 57 patients (43.5%), MRSA, in 21 patients (16%), and streptococcus (Group A, B, C, G) in 14 patients (10%). Less common organisms included acinetobacter baumanii, and klebsiella oxytoca.

## Conclusions

The high incidence of MRSA in our patient population is of concern, especially as the choice of antibiotics available to treat infections with this organism is slowly becoming reduced. Major areas of lack of knowledge in the care of patients with foot ulceration include the optimal duration of antibiotic treatment, both oral and parenteral, for infected ulcers and osteomyelitis in diabetic patients.

